# Who is More Likely to Use the Internet for Health Behavior Change? A Cross-Sectional Survey of Internet Use Among Smokers and Nonsmokers Who Are Orthopedic Trauma Patients

**DOI:** 10.2196/mental.7435

**Published:** 2017-05-30

**Authors:** Sam McCrabb, Amanda L Baker, John Attia, Zsolt J Balogh, Natalie Lott, Kerrin Palazzi, Justine Naylor, Ian A Harris, Christopher Doran, Johnson George, Luke Wolfenden, Eliza Skelton, Billie Bonevski

**Affiliations:** ^1^ School of Medicine and Public Health University of Newcastle Callaghan Australia; ^2^ Hunter Medical Research Institute University of Newcastle New Lambton Australia; ^3^ Department of General Medicine John Hunter Hospital New Lambton Heights Australia; ^4^ Department of Traumatology John Hunter Hospital New Lambton Heights Australia; ^5^ Whitlam Orthopaedic Research Centre Ingham Institute for Applied Medical Research Liverpool Hospital Liverpool Australia; ^6^ South Western Sydney Clinical School Faculty of Medicine University of New South Wales Liverpool Australia; ^7^ School of Human, Health and Social Sciences Central Queensland University Brisbane Australia; ^8^ Centre for Medicine Use and Safety Monash University Parkville Australia; ^9^ Hunter New England Population Health Wallsend Australia

**Keywords:** Internet, health, eHealth, health care, smoking, orthopedic trauma

## Abstract

**Background:**

eHealth presents opportunities to provide population groups with accessible health interventions, although knowledge about Internet access, peoples’ interest in using the Internet for health, and users’ characteristics are required prior to eHealth program development.

**Objective:**

This study surveyed hospital patients to examine rates of Internet use, interest in using the Internet for health, and respondent characteristics related to Internet use and interest in using the Internet for health. For patients who smoke, preferences for types of smoking cessation programs for use at home and while in hospital were also examined.

**Methods:**

An online cross-sectional survey was used to survey 819 orthopedic trauma patients (response rate: 72.61%, 819/1128) from two public hospitals in New South Wales, Australia. Logistic regressions were used to examine associations between variables.

**Results:**

A total of 72.7% (574/790) of respondents had at least weekly Internet access and more than half (56.6%, 357/631) reported interest in using the Internet for health. Odds of at least weekly Internet usage were higher if the individual was born overseas (OR 2.21, 95% CI 1.27-3.82, *P*=.005), had a tertiary education (OR 3.75, 95% CI 2.41-5.84, *P*<.001), or was a nonsmoker (OR 3.75, 95% CI 2.41-5.84, *P*<.001). Interest in using the Internet for health increased with high school (OR 1.85, 95% CI 1.09-3.15, *P*=.02) or tertiary education (OR 2.48, 95% CI 1.66-3.70, *P*<.001), and if household incomes were more than AUS $100,000 (OR 2.5, 95% CI 1.25-4.97, *P*=.009). Older individuals were less interested in using the Internet for health (OR 0.98, 95% CI 0.97-0.99, *P*<.001).

**Conclusions:**

Online interventions may be a potential tool for health care in this hospitalized population.

**Trial Registration:**

Australian New Zealand Clinical Trials Registry (ANZCTR): ACTRN12614001147673; https://www.anzctr.org.au/Trial/Registration/TrialReview.aspx?id=366829&isReview=true (Archived by WebCite at http://www.webcitation.org/6qg26u3En)

## Introduction

Worldwide rates of Internet access are high, with more than 3.5 billion people currently connected [1]. This reach is ever increased by mobile networks, which cover more than 90% of the globe, a reach forecasted to penetrate 71% of the global population by 2019 [2]. In Australia, 86% of households have Internet access, a rate which continues to increase with the development of the National Broadband Network [3,4]. Further, 3G and 4G networks increase this reach through mobile phone and tablet devices [5,6], making Internet-based programs more available. Using the Internet and technology for health, otherwise known as eHealth, is becoming more popular, with online programs used to deliver mental health care [7], change health behaviors [8], and deliver postsurgery rehabilitation programs [9]. eHealth programs are also attractive means of delivering interventions because they have a low cost per user [4,10], have the potential to reach people who may not have otherwise sought support [11], reduce stigma [4], and provide timely support when it is most needed [4]. Additionally, utilizing eHealth has been found to be acceptable to health care providers and patients [12,13].

Reviews on the effectiveness of eHealth interventions for health behavior change have found a mixed effect, stating that more research is needed in this area [14-17]. Potential factors contributing to this finding may include low uptake, adherence, or retention to online programs, with the format of online interventions regarded as an important factor in intervention effectiveness [18]. Therefore, consumer-based research to help understand patient-related factors likely to improve eHealth intervention use is important.

Within the hospital setting, previous research with people receiving treatment for orthopedic trauma has found that the receipt of health behavior change advice is low, despite patients’ interest in receiving such care [19,20]. Reasons for low levels of behavior change support include lack of time or appropriate knowledge [21-28]. eHealth interventions may be one way to address this lack of care by providing behavior change support with minimal staff involvement. Previous eHealth programs developed to promote postsurgery rehabilitation have been acceptable to patients [9]. Another potential benefit is that eHealth-delivered care can be continued postdischarge from hospital, a component of hospital interventions that has been found to improve effectiveness [29].

Rates of engagement in risky behaviors such as heavy alcohol use, cannabis use, and tobacco smoking have been found to be higher in the orthopedic trauma population than the general population [19,30,31]. Continued tobacco use can have devastating effects on recovery from surgery, such as increased risk of postoperative infection [32-34], wound and flap necrosis [35], and a decrease in the tensile strength of wounds [36]. The benefits of abstinence after surgery are clear, with a review of 20 studies finding significantly fewer complications in former smokers when compared to current smokers postoperation [32]. The benefits of abstinence from postoperative smoking may include better wound healing, shorter length of hospital admission, decreased risk of mortality [37], as well as reduced interaction with prescribed medications [38]. Similar to tobacco use, alcohol and cannabis use can have a negative impact on recovery from surgery such as vasoconstriction resulting in decreased blood flow and delays in healing as well as lung disorders, if smoked (cannabis) [39]; and increased risk of infection and impaired wound healing (alcohol) [40]. Therefore, understanding the rates of usage of substances following orthopedic trauma may be a potential health risk behavior that needs addressing.

Characterization of orthopedic trauma patients has previously found they are more often younger males who come from a lower socioeconomic status, partake in more risky behavior, and have a lower level of educational attainment [31]. Younger males [3,41-43], who are of a higher socioeconomic status [41,43] and have attained a higher level of education [6,41,44], are more likely to use the Internet for health; however, younger males are also less likely to seek health care [45-48]. Therefore, online programs may provide an option to increase the care they receive during admission and postdischarge.

Previous research suggests that 75% to 92% of orthopedic trauma patients use the Internet [49-51]. Between 45% and 58% reported using the Internet for health information [49,50] and the majority used the Internet at home [51]. Further, using the Internet for postsurgery follow-up has been found acceptable by patients in this population [52,53]. This suggests that online health behavior change interventions may be well received by patients.

Although rates of access to the Internet are high, there is limited information about interest in using the Internet for health and no knowledge regarding the characteristics of patients more likely to use the Internet for health. Such information helps develop eHealth interventions that reach and engage the highest number of the target population. Because previous rates of current tobacco smoking, alcohol, and cannabis use are high among orthopedic trauma patients, an online program may be a potential option to address these risky health behaviors. Therefore, this study of hospitalized orthopedic trauma patients has a number of aims:

1. Describe rates of Internet use, device use, and interest in using the Internet for health;
2. Examine patient characteristics associated with frequent (at least weekly) Internet use and interest in using the Internet for health; and
3. For patients who smoke, measure patient preferences for types of smoking cessation programs for use at home and while in hospital.

## Methods

### Design and Setting

An online cross-sectional survey was conducted with orthopedic trauma inpatients in two public hospitals in New South Wales, Australia. Surveys were conducted between April 2015 and September 2016. Ethics approval was obtained from Hunter New England Human Research Ethics Committee (approval number: 14/02/19/4.04), with site approval from the University of Newcastle Human Research Ethics Committee (approval number: H-2014-0081) and the South West Sydney Human Research Ethics Committee (approval number: HREC/14/HNE/46; SSA/14/LPOOL/191).

### Participants

Patients were eligible if they had been admitted to hospital with a fracture, were aged between 18 and 80 years, and were able to read and comprehend written English. Patients judged incapable of providing consent by the research personnel were not approached to take part.

Patients were approached during admission by a research assistant (RA) to participant in an online health survey of orthopedic trauma patients. The RAs were provided daily with a list of new orthopedic trauma admissions from a research nurse. New admissions were approached consecutively by the RAs who assessed eligibility and gained informed consent. If an individual was too sick to be seen or was busy with medical staff on the day they were first approached, they were approached the following day. All participants were provided with a survey number to de-identify their results.

### Measures

Existing validated items were used or adapted where possible [54-57]. Survey items are provided in Multimedia Appendix 1 and form part of a larger survey, which took respondents approximately 15 minutes to complete.

#### Participant Demographics

Respondent characteristics, such as gender, age, country of birth, indigenous status, marital status, education, main source of income, household income, and health insurance type, were all assessed.

#### Smoking Status and Smoking-Related Variables

Current smoking status was determined for all respondents using the questions: “Do you currently smoke tobacco?” (yes, daily; yes, at least once a week; yes, less than once a week; no, not at all) and “Have you smoked at least 100 cigarettes or a similar amount of tobacco in your life?” (yes; no; not sure) [57].

#### Alcohol Use

The Alcohol Use Disorder Identification Test (AUDIT-C) [54] was used to determine alcohol usage. Scoring for the AUDIT-C ranges from zero to 12 with cut-offs of three for females (sensitivity: 66%-73%; specificity: 91%-94%) [58,59] and four for males used to indicate heavy drinking (sensitivity: 86%; specificity: 72%-89%) [54,59].

#### Cannabis

Recent cannabis use was measured using a single item based on questions asked in the Opiate Treatment Index (OTI) [55]. Respondents were asked: “Have you used cannabis (marijuana, dope, grass, hash, pot) in the last 30 days?” (yes; no).

#### Internet-Related Questions

Individuals were asked questions relating to their use of the Internet [56] and if they would use the Internet to improve their health. Respondents were asked: (1) “In the last 12 months, how often have you accessed the Internet?” (every day; a few times per week; about once a week; less than once a week; not at all); (2) “In the last 12 months, did you access the Internet through any of the following? Computer (desktop or laptop), smartphone (eg, iPhone or Android), tablet (eg iPad), a device not owned by you (eg, a friend’s smartphone, library or work computer)” (yes; no); (3) “Would you use the Internet to help improve your health?” (yes; no); and (4) “Do you have a computer with Internet access at home?” (yes; no).

#### Interest in Quit-Smoking Programs

Individuals who indicated that they were current tobacco users and had the Internet at home were also asked questions related to their interest in using a quit-smoking program on their computer at home and what type of quit-smoking program they would be interested in using while in hospital. Respondents were asked: “Would you use a quit-smoking program on your computer at home?” (definitely yes; maybe; unlikely; no) and “If available, what types of quit-smoking programs would you be interested in using while in hospital? DVD/television, printed booklet, telephone counseling, mobile phone text messaging, Internet program, face-to-face counseling” (yes; no).

### Analysis

All data were stored on secure servers at the University of Newcastle and were exported into STATA version 13 (StataCorp LP, College Station, TX, USA) for analysis.

Descriptive statistics of participant sociodemographics are presented as numbers and percentages for categorical variables and means (standard deviation) or median (interquartile range) for continuous variables, depending on distribution of the data. Binary logistic regressions were used to examine the associations between age, gender, income, and education with at least weekly Internet use and with interest in using the Internet for health. At least weekly Internet access was determined by combining “every day,” “a few times per week,” and “about once a week.” Variables entered in the model were selected a priori and included factors that have been previously associated with tobacco smoking in medically ill and general populations: age, gender, country of birth, education, marital status, and household income [60-62].

Adjusted odds ratios with 95% confidence intervals and *P* values were calculated for variables in the models. Significance was determined at *P*<.05. Collinearity of variables related to weekly Internet use and interest in using the Internet for health were checked using variance inflation factors (VIFs). No variables were found to be collinear, with all VIFs less than two.

## Results

A total of 1708 orthopedic trauma admissions occurred during the study period, of which 1128 were approached and 819 subsequently agreed to participate in the survey (72 refused, 103 were too ill to participate, and 134 were not eligible; response rate: 72.61%). Some respondents dropped out during survey completion due to fatigue. A total of 803 individuals completed the survey (completion rate: 98.0%). Of these individuals, 175 (21.8%) identified as current tobacco users and form the subpopulation analyzed in this paper. A total of 173 current smokers completed the survey (completion rate: 98.9%). Due to the format and branching of survey questions, not all smokers answered the same questions (eg, individuals who did not own a computer at home were not asked if they would use a smoking cessation program on their computer at home).

### Patient Demographics

Table 1 contains a summary of patient demographic information.

### Rates of Internet Use and Type of Technology

Table 2 shows the rates of Internet use, access to the Internet at home, and types of technology used to access the Internet for the sample and by smoking status.

### Interest in Using Technology for Health

Overall rate of agreement in using the Internet to improve health for the whole population was 56.6% (357/631) and 53.5% (76/489) for current tobacco users (Table 2).

### Interest in Smoking Cessation Programs

Looking specifically at interest in smoking cessation programs for current tobacco users, Table 3 indicates that the majority (47.1%, 49/104) of current tobacco smokers would “definitely yes” or “maybe” use a quit-smoking program on their computer at home.

**Table 1:** Sociodemographic characteristics of the sample.

Sociodemographic characteristic and response options		Total (N=819)	
**Gender, n (%)**			
	Male		489 (59.7)
	Female		330 (40.3)
**Age (years)**			
	Mean (SD)		50.5 (17.7)
	Median (IQR)		54 (35-66)
**Country of birth, n (%)**			
	Australia		688 (84.1)
	Other		130 (15.9)
**Indigenous status, n (%)**			
	Aboriginal		39 (4.8)
	Torres Strait Islander		4 (0.5)
	Both Aboriginal and Torres Strait Islander		1 (0.1)
	Neither		774 (94.6)
**Marital status, n (%)**			
	Married		336 (41.1)
	De facto/living with partner		135 (16.5)
	Separated/divorced		86 (10.5)
	Single		204 (24.9)
	Widowed		57 (7.0)
**Education, n (%)**			
	No formal education		4 (0.5)
	Primary school		17 (2.1)
	High school (7-10)		241 (29.5)
	High school (11-12)		131 (16.0)
	TAFE or trade		311 (38.0)
	University		114 (13.9)
**Main source of income, n (%)**			
	Paid employment (either full or part time)		427 (52.2)
	Government pension or benefit		293 (35.8)
	Family member		29 (3.6)
	Savings or retirement funds		37 (4.5)
	Other		32 (3.9)
**Household income (AUS$), n (%)**			
	<$20,000 per year		80 (9.8)
	$20,000-$50,000 per year		213 (26.1)
	$51,000-$70,000 per year		129 (15.8)
	$71,000-$100,000 per year		92 (11.3)
	>$100,000 per year		101 (12.4)
	Prefer not to state		200 (24.5)
**Insurance type, n (%)**		(n=815)	
	Comprehensive private health insurance		253 (31.0)
	Private health insurance-without extras		51 (6.3)
	Private health insurance-extras only		34 (4.2)
	Department of Veteran’s Affairs white or gold card		11 (1.4)
	Health care concession card		243 (29.8)
	None of these		223 (27.4)
**Smoking status, n (%)**		(n=803)	
	Daily smoker		157 (19.6)
	Occasional smoker		18 (2.2)
	Exsmoker		235 (29.3)
	Nonsmoker		393 (48.9)
**AUDIT-C, n (%)**		(n=795)	
	Nondrinker		185 (23.3)
	Non-heavy drinker		198 (24.9)
	Heavy drinker		412 (51.8)
**Cannabis use last 30 days, n (%)**		(n=811)	
	No		732 (90.3)
	Yes		79 (9.7)

**Table 2:** Rates of Internet use, types of technology used, and interest in using the Internet for health for the sample.

Question and response options		Smoker, n (%)	Nonsmoker, n (%)	Total, n (%)^a^
**Internet access last 12 months**		(n=173)	(n=617)	(N=790)
	Every day	82 (47.4)	355 (57.5)	437 (55.3)
	A few times per week	19 (11.0)	92 (14.9)	111 (14.1)
	About once a week	9 (5.2)	16 (2.6)	26 (3.2)
	Less than once a week	11 (6.4)	29 (4.7)	40 (5.1)
	Not at all	52 (30.1)	125 (20.3)	177 (22.4)
**Devices used to access the Internet last 12 months**		(n=173)	(n=617)	(n=790)
	Computer (desktop or laptop)	87 (50.3)	426 (69.0)	513 (64.9)
	Smartphone	113 (65.3)	418 (67.9)	531 (67.3)
	Tablet	48 (27.8)	222 (36.0)	270 (34.2)
	A device not owned by you	42 (24.3)	176 (28.6)	218 (27.6)
**Do you have a computer at home with Internet access?**		(n=153)	(n=554)	(n=707)
	Yes	99 (64.7)	423 (76.4)	522 (73.8)
**Would you use the Internet to improve your health?**		(n=489)	(n=142)	(n=631)
	Yes	76 (53.5)	281 (57.5)	357 (56.6)

^a^ Respondents with available data.

**Table 3:** Interest in smoking cessation program (current smokers only, n=175).

Question and response options		n (%)
**Would you use a quit-smoking program on your computer at home? (n=104)^a^**		
	Definitely yes	18 (17.3)
	Maybe	31 (29.8)
	Unlikely	16 (15.4)
	No	39 (37.5)
**If available, what type of quit-smoking program used while in hospital? (n=159)^a^**		
	DVD/television	37 (22.6)
	Printed booklet	42 (26.4)
	Telephone counseling	35 (22.0)
	Mobile phone text messaging	37 (23.3)
	Internet program	46 (28.9)
	Face-to-face counseling	52 (32.7)
	None of these	73 (45.9)

^a^ Current smokers with available data.

### The Relationship Between Age, Gender, Smoking Status, and Substance Use With at Least Weekly Internet Usage

The odds of having at least weekly Internet access was 1.08 times lower per year older (95% CI 0.90-0.93, *P*<.001). The odds of at least weekly Internet access were found to be 2.21 times higher if the respondent was born overseas when compared to Australian born (95% CI 1.27-3.82, *P*=.005), 3.75 times higher if the individual had a tertiary education (95% CI 2.41-5.84, *P*<.001), 3.51 times higher if the individual was a nonsmoker (95% CI 2.03-6.09, *P*<.001), and 1.90 times higher for a heavy drinker when compared to a nondrinker (95% CI 1.15-3.14, *P*=.01) (Table 4).

### The Relationships Between Age, Gender, and Substance Use With Interest in Using the Internet for Health

The odds of being interested in using the Internet to improve health was 1.02 times lower for each year older (95% CI 0.97-0.99, *P*<.001). Conversely, the odds of being interested in using the Internet to improve health was 2.48 times higher for individuals if they had a tertiary education (95% CI 1.66-3.07, *P*<.001), 1.85 times higher if the individual had a high school education (95% CI 1.09-3.15, *P*=.02), and 2.5 times higher if the participant had a household income of more than AUS $100,000 (95% CI 1.25-4.97, *P*=.009) (Table 5).

## Discussion

A minimum of at least weekly Internet usage was slightly lower in this sample of orthopedic trauma patients than the national average (72.5% vs 86%) [6]. As expected, age and education were both found to be associated with at least weekly Internet access. Country of birth, educational attainment, smoking status, and alcohol consumption were also found to be significant predictors of Internet access. These are important patient characteristics to note because rates of substance use and factors around their use and treatment often differ between cultures [63], a point which may need considering in the development of any eHealth interventions for substance use within this population. Further, when examining Internet use by current smoking status, the percentage of at least weekly access dropped to 63.6%. This may indicate that an online smoking cessation program may only benefit those individuals who are younger and who have a greater level of educational attainment. This is important because the development of an online program may segregate a portion of the population who are unable to access the Internet postdischarge (if hospitals provide devices and Internet access to use during admission). Further, this may reflect the lower socioeconomic status of trauma patients [31] and current tobacco users [64], with younger age and a higher level of education attainment related to higher rates of Internet usage [3,41,44]. Differences in access to the Internet may deepen the digital divide in receipt of care, with age and education [44] found to be associated with access to eHealth interventions. However, previous research suggests the digital divide to be a myth in the orthopedic trauma population [51], although results from this study may suggest otherwise. Therefore, the possibility of a digital divide should be acknowledged during intervention development and implementation, with alternate care designed for those who may not have access.

**Table 4:** Logistic regression of the associations with at least weekly Internet use for the whole sample (N=805).

Variable		At least weekly Internet use, n (%)	Crude OR (95% CI)	*P*	AOR^a^ (95% CI)	*P*
Age			0.93 (0.92-0.94)	<.001	0.92 (0.90-0.93)	<.001
**Gender**				.001		.50
	Male	378 (78.1)	Ref		Ref	
	Female	208 (64.8)	0.52 (0.38-0.71)		1.16 (0.75-1.79)	
**Country of birth**				.25		.005
	Australia	486 (72.0)	Ref		Ref	
	Other	100 (76.9)	1.30 (0.83-2.02)		2.21 (1.27-3.82)	
**Education**				<.001		<.001
	No formal/primary school/high school (7-10)	130 (50.4)	Ref		Ref	
	High school (11-12)	97 (75.2)	2.98 (1.87-4.77)	<.001	1.37 (0.77-2.46)	.29
	TAFE trade/university	359 (85.9)	5.99 (4.15-8.66)	<.001	3.75 (2.41-5.84)	<.001
**Marital status**				.005		.07
	Married/de facto	353 (76.6)	Ref		Ref	
	Single/widowed/ separated/divorced	233 (67.7)	0.64 (0.47-0.88)		0.67 (0.44-1.03)	
**Household income (AUS$)**				<.001		.21
	<$50,000	178 (61.6)	Ref		Ref	
	$51,000-$100,000	169 (77.2)	2.11 (1.42-3.13)	<.001	1.26 (0.75-2.11)	.38
	> $100,000	93 (93.0)	8.28 (3.71-18.51)	<.001	2.45 (0.98-6.13)	.06
	Prefer not to state	146 (74.1)	1.79 (1.20-2.66)	.004	1.10 (0.66-1.83)	.71
**Smoker**				.003		<.001
	Current	110 (63.6)	Ref		Ref	
	Nonsmoker	463 (75.0)	1.72 (1.20-2.47)		3.51 (2.03-6.09)	
**Cannabis use**				.51		.63
	No	526 (72.5)	Ref		Ref	
	Yes	60 (76.0)	1.20 (0.70-2.06)		0.84 (0.40-1.73)	
**Alcohol consumption**				<.001		.04
	Nondrinker	100 (54.6)	Ref		Ref	
	Non-heavy drinker	148 (75.5)	2.56 (1.65-3.96)	<.001	1.67 (0.96-2.90)	.07
	Heavy drinker	324 (79.0)	3.13 (2.15-4.55)	<.001	1.90 (1.15-3.14)	.01

^a^ Model adjusted for age, gender, country of birth, marital status, household income, current smoking status, cannabis use, and alcohol consumption. AOR: adjusted odds ratio.

**Table 5:** Logistic regression of associations with interest in using the Internet for health for the whole sample (n=644).

Variable		Interested in using Internet for health, n (%)	Crude OR (95% CI)	*P*	AOR^a^ (95% CI)	*P*
Age			0.98 (0.97-0.99)	<.001	0.98 (0.97-0.99)	<.001
**Gender**				.81		.36
	Male	226 (57.2)	Ref		Ref	
	Female	140 (56.2)	0.96 (0.70-1.32)		1.19 (0.82-1.75)	
**Country of birth**				.82		.45
	Australia	304 (57.0)	Ref		Ref	
	Other	62 (55.9)	0.95 (0.63-1.44)		1.20 (0.75-1.90)	
**Education**				<.001		<.001
	No formal/primary school/high school (7-10)	82 (38.7)	Ref		Ref	
	High school (11-12)	63 (60.6)	2.44 (1.51-3.94)	<.001	1.85 (1.09-3.15)	.02
	TAFE Trade/University	221 (67.6)	3.27 (2.28-4.69)	<.001	2.48 (1.66-3.70)	<.001
**Marital status**				.54		.76
	Married/de facto	213 (57.9)	Ref		Ref	
	Single/widowed/ separated/divorced	153 (55.4)	0.91 (0.66-1.24)		1.06 (0.73-1.54)	
**Household income (AUS$)**				<.001		.001
	<$50,000	121 (51.7)	Ref		Ref	
	$51,000-$100,000	98 (63.6)	1.63 (1.08-2.48)	.02	1.50 (0.94-2.40)	.09
	>$100,000	64 (82.1)	4.27 (2.27-8.04)	<.001	2.50 (1.25-4.97)	.009
	Prefer not to state	83 (46.6)	0.82 (0.55-1.21)	.31	0.73 (0.47-1.12)	.15
**Smoker**				.40		.67
	Current	76 (53.5)	Ref		Ref	
	Nonsmoker	281 (57.5)	1.17 (0.81-1.71)		1.10 (0.70-1.73)	
**Cannabis use**				.96		.86
	No	330 (56.8)	Ref		Ref	
	Yes	36 (57.1)	1.01 (0.60-1.72)		1.06 (0.57-1.95)	
**Alcohol consumption**				.04		.63
	Nondrinker	69 (47.6)	Ref		Ref	
	Non-heavy drinker	91 (58.3)	1.54 (0.98-2.43)	.06	1.26 (0.76-2.08)	.37
	Heavy drinker	198 (60.2)	1.66 (1.12-2.47)	.01	1.20 (0.77-1.87)	.41

^a^ Model adjusted for age, gender, country of birth, marital status, household income, current smoking status, cannabis use, and alcohol consumption. AOR: adjusted odds ratio.

Just over half the respondents indicated that they would be interested in using the Internet for health, with interest in using the Internet for health found to be associated with younger age, higher education, and higher household income. Other research has found greater use of the Internet for health by users with higher socioeconomic status [65] and this too may contribute to the potential for a digital divide in this population. Alternatively, patients may simply be unaware of what eHealth programs are and what they could deliver. In a large mental health survey, attitudinal resistance toward Internet-based interventions was cited as a possible explanation for lack of interest in using the Internet for heath [66]. Patient education of eHealth programs may counter this effect [67].

Tobacco smoking in particular is a serious threat to the recovery and health of orthopedic trauma patients. When respondents who were current tobacco users were asked what types of quit-smoking programs they would prefer to use while in hospital, an Internet program was supported, second only to face-to-face counseling. Given there are a multitude of barriers to the delivery of face-to-face smoking cessation counseling, these results suggest online programs may be well received by patients who smoke. Continuing to address smoking in the orthopedic trauma population has the added benefit of reducing the overall health-related costs, both to the individual and the health care system.

### Limitations

The main limitation of this study is that the sample was recruited from two hospitals and the results are not generalizable to other hospital population patients groups (eg, cardiac patients) because characteristics between different medical groups differ, with orthopedic trauma patients being usually younger, risk-taking males [31].

### Implications

These results suggest that access to the Internet and interest in using the Internet for health may be acceptable to some orthopedic trauma patients. Patients who use tobacco reported interest in receiving additional support to quit during admission through eHealth interventions. The provision of care through eHealth interventions may change the landscape of the health care environment in primary settings because it may provide a form of care that is acceptable to patients, while addressing some of the limitations to the provision of care by staff (ie, lack of appropriate knowledge or skills [24-27], time constraints, and lack of resources) [23,27,28]. Although no health behavior change intervention programs for orthopedic trauma patients are known to the authors, online programs have been implemented in other medical populations [68,69], with mixed effects found. Further, smoking cessation-specific eHealth interventions have been implemented in the general population and have been found to be effective [70-72].

Notably, a Cochrane review of smoking cessation for hospitalized patients found interventions that are more intensive and contain at least one month follow-up after discharge from hospital are effective at increasing cessation following hospital admission [29]. Therefore, online programs could be used postdischarge, providing the recommended follow-up support.

### Conclusions

Online health programs appear to be of interest to orthopedic trauma patients. In particular, development of online programs to assist patients who smoke to quit smoking during their hospitalization and postdischarge may be suitable for this population. An online program could be acceptable to the majority of trauma patients that are current tobacco users who desire more help to quit. The development of novel approaches to providing health care may change the landscape of the primary health setting.

## Appendix

Multimedia Appendix 1 Supplementary file.

## Abbreviations

AUDIT-C: Alcohol Use Disorder Identification Test
OTI: Opiate Treatment Index
RA: research assistant
VIF: variance inflation factor
