# Supplementary material for: Who is More Likely to Use the Internet for Health Behavior Change? A Cross-Sectional Survey of Internet Use Among Smokers and Nonsmokers Who Are Orthopedic Trauma Patients
Source: JMIR Ment Health. 2017 May 30;4(2):e18. doi: 10.2196/mental.7435 (PMC5470009; doi:10.2196/mental.7435)
Supplement: Multimedia Appendix 1 [file mental_v4i2e18_app1.pdf]

Supplementary file

Survey question	Response options
Smoking status and smoking related variables [1].	
Do you currently smoke tobacco?	Yes, daily<br>Yes, at least once a week<br>Yes, less than once a week<br>No, not at all
Have you smoked at least 100 cigarettes or a similar amount of tobacco in your life?	Yes<br>No<br>Not sure
Alcohol use [2]	
How often do you have a drink containing alcohol?	Never<br>Monthly or less<br>2-4 times a month<br>2-3 times a week<br>4 or more times a week
How many standard drinks containing alcohol do you have on a typical day?	0 drinks<br>1 or 2 drinks<br>3 or 4 drinks<br>5 or 6 drinks<br>7 to 9 drinks<br>10 or more drinks
How often have you had four or more standard drinks on one occasion?	Never<br>Less than monthly<br>Monthly<br>Weekly<br>Daily or almost daily
Cannabis use [3]	
Have you used cannabis (marijuana, dope, grass, hash, pot) in the last 30 days	Yes<br>No
Internet related questions [4]	
In the last 12 months, how often have you accessed the internet?	Every day<br>A few times per week<br>About once a week<br>Less than once a week<br>Not at all
In the last 12 months, did you access the internet through any of the following?*<br>Computer (desktop or laptop)<br>Smart phone (e.g. iPhone or Android)<br>Tablet (e.g. iPad)<br>A device not owned by you (e.g. a friend's smartphone, library or work computer)	Yes

	No
Would you use the internet to help improve your health?	Yes
	No
Do you have a computer with internet access at home?	Yes
	No
Interest in quit smoking programs^	
Would you use a quit smoking program on your computer at home?	Definitely yes
	Maybe
	Unlikely
	No
If available, what types of quit smoking programs would you be interested in using while in hospital?#	Yes
DVD/television	
Printed booklet	
Telephone counselling	
Mobile phone text messaging	
Internet program	
Face-to-face counselling	
	No

* Individuals were asked to indicate Yes or No to each computer, smart phone, tablet etc. for this question.

^ Only current tobacco users were asked these question.

Individuals were asked to indicate Yes or No to each DVD/Television, printed booklet, telephone counselling etc. for this question.

References

1. Mullins R, Borland R. Changing the way smoking is measured among Australian adults: a preliminary investigation of Victorian data. Quit evaluation studies 1998;9:163-73.
2. Bush K, Kivlahan DR, McDonell MB, Fihn SD, Bradley KA. The AUDIT alcohol consumption questions (AUDIT-C): an effective brief screening test for problem drinking. Ambulatory Care Quality Improvement Project (ACQUIP). Alcohol Use Disorders Identification Test. Arch Intern Med 1998;158:1789-95. PMID: 9738608
3. Darke S, Ward J, Hall W, Heather N, A W. The Opiate Treatment Index (OTI) Researcher's Manual. Sydney: National Drug and Alcohol Research Centre, 1991 Contract No.: 11.
4. Klein B, White A, Kavanagh D, Shandley K, Kay-Lambkin F, Proudfoot J, Drennan J, Connor J, Baker A, Young R. Content and functionality of alcohol and other drug websites: results of an online survey. J Med Internet Res 2010;12:e51. PMID: 21169168
